# Choice in maternity care: associations with unit supply, geographic accessibility and user characteristics

**DOI:** 10.1186/1476-072X-11-35

**Published:** 2012-08-20

**Authors:** Hugo Pilkington, Béatrice Blondel, Nicolas Drewniak, Jennifer Zeitlin

**Affiliations:** 1 INSERM, UMRS953, IFR69, Epidemiological Research Unit on Perinatal Health and Women’s and Children’s Health; UPMC Univ Paris6, Paris, France; 2 Département de Géographie, Université Paris 8 Vincennes – Saint Denis, Paris, France

**Keywords:** Health services accessibility, Distance, Hospital planning, Perinatal care

## Abstract

**Background:**

Despite national policies to promote user choice for health services in many European countries, current trends in maternity unit closures create a context in which user choice may be reduced, not expanded. Little attention has been paid to the potential impact of closures on pregnant women’s choice of maternity unit. We study here how pregnant women’s choices interact with the distance they must travel to give birth, individual socioeconomic characteristics and the supply of maternity units in France in 2003.

**Results:**

Overall, about one-third of women chose their maternity units based on proximity. This proportion increased steeply as supply was constrained. Greater distances between the first and second closest maternity unit were strongly associated with increasing preferences for proximity; when these distances were ≥ 30 km, over 85% of women selected the closest unit (revealed preference) and over 70% reported that proximity was the reason for their choice (expressed preference). Women living at a short distance to the closest maternity unit appeared to be more sensitive to increases in distance between their first and second closest available maternity units. The preference for proximity, expressed and revealed, was related to demographic and social characteristics: women from households in the manual worker class chose a maternity unit based on its proximity more often and also went to the nearest unit when compared with women from professional and managerial households. These sociodemographic associations held true after adjusting for supply factors, maternal age and socioeconomic status.

**Conclusions:**

Choice seems to be arbitrated in both absolute and relative terms. Taking changes in supply into consideration and how these affect choice is an important element for assessing the real impact of maternity unit closures on pregnant women’s experiences. An indicator measuring the proportion of women for whom the distance between the first and second maternity unit is greater than 30 km can provide a simple measure of choice to complement indicators of geographic accessibility in evaluations of the impact of maternity unit closures.

## Background

Providing pregnant women with a choice of maternity services has recently become a policy goal in France and other European countries and is a high priority demand of user associations [1,2]. Yet current trends in maternity unit closures in Europe create a context in which user choice may be reduced, not expanded [3-6]. Maintaining and adequate supply of maternity services, equity in choice as well as high standards of quality of care in remote rural areas is also a concern in other developed countries [7-10]. The debate on the consequences of maternity unit closures has focused primarily on the spatial accessibility of services and less attention has been paid to their potential impact on pregnant women’s choice of maternity unit. In a previous paper, we studied the impact of maternity unit closures on the accessibility of maternity services between 1998 and 2003 when 20% of France’s maternity units shut down [11]. This paper found that spatial accessibility was preserved for most women, meaning that they did not have increased travel time to get to the maternity unit where they delivered. In fact, travel times appeared to decrease for some women despite a reduction in supply. These counterintuitive results suggested that maternity unit closures may modify choice criteria and increase selection of maternity units because of their geographic proximity.

But just how does proximity influence choice? Numerous factors can contribute to an individual woman's choice of place to give birth. Adequacy, abundance and proximity of supply all play a part in the decision-making process [12]. Women can also select to go to the closest maternity unit for reasons other than their preference for proximity or less travel time. The closest maternity unit can have a great reputation, be recommended by a friend or have a specific technical expertise.

Our aim in this paper is to study how pregnant women’s choices interact with the distance they must travel to give birth and the supply of maternity services. By analyzing women’s preferences for proximity, we seek to establish how proximity is valued by the population when choice is not constrained (in situations of abundant supply) and how this evolves as supply characteristics become more constrained. This approach also allows us to identify the supply characteristics most strongly associated with changes in preferences: how context (supply and area-based characteristics) impacts choice of maternity unit and the reasons women give for making their choices. We further study how individual social and demographic characteristics affect these choice thresholds, in particular what lies behind the decision to go to the closest maternity unit or to travel longer to give birth. An underlying aim is to develop an indicator of choice based on data from routine surveys in France or birth registers, which are available in most countries, that can be monitored as maternity services are restructured.

### Choice as a dimension of access

There is now a wide body of literature on access to healthcare. In particular, studies have stressed the importance of an adequate amount of supply as well as an acceptable distance the individual must travel to reach a healthcare facility [13-17]. Other dimensions of access have focused on the individual's choice process.

Choice may be thought of as the opportunity or privilege of choosing freely out of a range of options available to an individual. Choice is also a dimension that explains revealed accessibility (what people actually did, as opposed to potential accessibility) – a tradeoff between need, service opportunities and individual and/or contextual determinants. Age, education, parity, country of birth and other cultural factors (language, immigration status, healthcare coverage) and to a lesser extent, income, have all been associated with choice in previous studies in Canada, France and the UK and other countries [18-22]. As such, choice is a dimension closely related to – or part of – the process shaping individual accessibility to healthcare, as defined by Penchansky and Thomas [23] alongside availability, accommodation, affordability and acceptability.

A particular area where the notion of choice comes into play lies within spatial accessibility, or the distance an individual must travel for care. It has long been accepted that the further a particular service is situated from an individual, the less likely the person is to access that service. In geography, this has been described by the distance decay effect, whereby increased distance between consumers of a particular health service and medical facilities results in a weakening of the provider–consumer link [24]. This builds on applications of Jarvis’ law to accessibility to healthcare facilities (other than Jarvis' initial work on mental health) [25]. Distance is a major determinant of hospital choice in many areas of healthcare and has been studied in obstetrics too [15,19,26-28]. As such, low supply and longer travel distances penalize access – the ‘fit’ between the patient and the healthcare system – in a number of ways.

Yet distance is not the only factor involved in poor access to healthcare. It represents a constituting factor in the set of possibilities bounded by constraints an individual is faced with when needing to resort to care. In other words, the distance a woman must travel to a maternity unit interacts with other dimensions a woman considers when choosing where to deliver, i.e. distance forms a part of that person’s choice set, following work by Le Grand [29]. In many of these studies, distance to care acts as a surrogate measure for key dimensions in accessibility, such as travel time or travel cost alongside familiarity or personal preference for a given healthcare service.

### The French context

In France, women are free to choose where they wish to deliver – there are no restrictions (even though this may not apply to some specific situations, such as the 1% of women who have no health insurance coverage [30] and women with high risk pregnancies who must deliver in tertiary maternity units). Previous work suggests that supply may affect the reasons that women select their maternity units [31]. Choice is more complex in areas where there are many units, for instance urban or peri-urban areas [21,32,33]. Some research specific to France also suggests that women prefer more specialised centres [34] although mothers in very rural areas may tend to base their choice on proximity alone [31]. In addition, very few women in France choose to deliver at home [35]. This leads us to be interested in comparing women’s choices in situations with different supply characteristics in this setting.

### Conceptualizing choice

Most studies of “revealed accessibility” analyze choice as a function of need and service opportunities mediated by individual characteristics and apply spatial models to user choice [15] using some variant of gravity-based models under the assumptions that the attraction of a hospital is inversely related to travel time and that non-spatial determinants mediate this inverse-care law. Yet few studies have considered choice as a result of an arbitration process by the user according to contextual-level (i.e. area-level) and individual determinants.

One way to operationalize this concept is to compare women’s actual choices and their stated reasons for choices in situations where choices are abundant and those where there is less or, at an extreme, no choice. For maternity care, a choice is only possible if there are two maternity units within a reasonable distance from home. What constitutes a “reasonable distance” is, however, a dimension of personal preference that is difficult to capture. It is closely related to acceptability, a dimension of accessibility that should be taken into account in any study of access to healthcare. Women with only one maternity unit close by would be forced to choose this unit. In contrast, in a situation with abundant supply, some women would select the maternity unit closest to their home because of a preference for proximity and reduced travel time or because the closest unit had other characteristics that they value, while others with different preferences would go farther for care.

For these reasons, in this analysis we compare women’s observed and stated preferences for proximity as a way of assessing the impact of distance, supply and other characteristics on choice. Specifically, our research questions are:

1)How does context influence user choice? Context here refers to a given supply of maternity units in a given region at some distance from the woman’s residence – it is a contextual level determinant in accessibility to health care;

2)How do user characteristics affect the relationship between context and choice?

3)How may an indicator of choice be incorporated into an assessment of the impact of changes in access to health services when the supply of maternity services is restructured and in particular when maternity units are closed down?

## Results

Table 1 presents expressed preferences for proximity by supply factors. Overall, 36.4% of women in our National Perinatal Survey (NPS) sample declared they selected their maternity unit based on proximity. Women chose their maternity unit based on proximity slightly more often when they were living less than 5 km from their closest unit (37.7%), than when the closest maternity unit was farther away (33.5% for women living at 30 km or more from their closest units). Greater distances between the first and second closest maternity unit were strongly associated with increasing preferences for proximity. Women living in areas where their two closest maternity units were separated by <1 km chose their unit based on proximity 25% of the time versus 70% when there was a 30 km or more distance between their 2 closest units. Having more than 2 units in a 15 km radius did not appear to have an impact for expressed preferences. For women’s observed behaviour, patterns are similar, although the revealed preference for proximity is overestimated for women whose first and second maternity units are in the same commune for reasons described in the methods section.

**Table 1:** Supply factors associated with expressed and revealed preference

**Supply factors**	**Reason for choice is proximity (NPS**^**1**^**)**		**Closest unit was chosen (VS**^**2**^**)**	
	**N**	**%**	**N**	**%**
	9657	36.4	736358	62.1
Distance to the closest maternity unit (km)				
<5	4405	37.7	337566	62.9
5-14	2571	36.8	193939	61.9
15-29	2049	34.2	153919	62.3
30+	630	33.5	50934	55.9
p		0.0197		<0.001
Distance between 1st and 2nd closest maternity unit (km)				
0	3891	25.1	287061	78.3^3^
1-4	2089	31.8	165762	32.8
5-14	2028	42.5	153782	49.4
15-29	1051	56.9	83828	74.7
30+	596	69.8	45925	85.5
p		<0.001		<0.001
Units in a 15 km radius				
0	2680	34.0	204786	60.7
1	1727	55.7	130133	73.2
2	1356	31.6	99009	79.7
3	1045	25.1	71883	79.0
4-9	1167	31.0	93953	54.9
10+	1682	35.3	136594	36.7
p		<0.001		<0.001

^1^NPS: National Perinatal Survey.

^2^VS: Vital Statistics Registry.

^3^When units are located in the same commune, the distance between them is computed as zero; units in the same commune are all considered to be closest.

**Figure 1:**
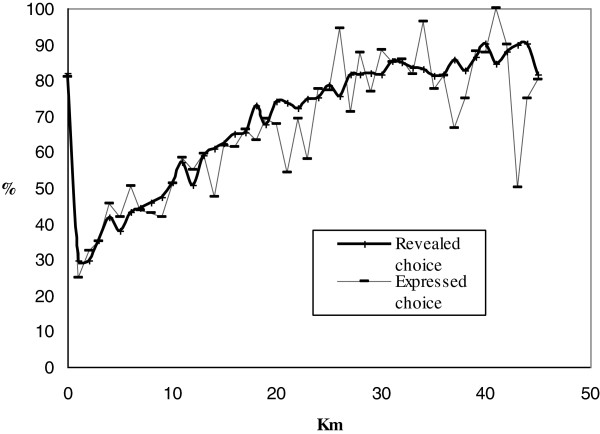
**Percentage of women who chose and who stated choosing closest maternity unit by distance between 1**^**st **^**and 2**^**nd **^**maternity unit.** The plot shows the percentage of women who declared that proximity was the reason for their choice of maternity unit and the percentage of women who actually chose their closest maternity unit.

Figure 1 provides more detail on the association of distance between the first and second unit and preferences for proximity using the NPS and Vital Statistics (VS) data. Both expressed and revealed preference for proximity increases as the distance between the first and second maternity unit increases. Starting at about 30 km, the choice of the farther unit is rare and evens out. It should be noted that at 0 km, the upturn of the curve reflects the inability of our data to distinguish between two units located in the same commune and thus clearly overstates the revealed preference for proximity in the sample; extrapolating from the observed pattern would place revealed preference in these situations at about 30%.

**Figure 2:**
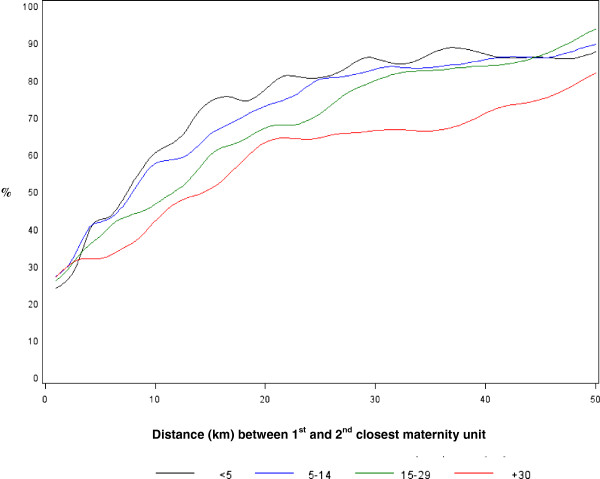
**Choice of closest maternity unit by distance to closest maternity unit and increasing distance between 1**^**st **^**and 2**^**nd **^**closest unit.** This figure plots the percentage of women whose chose the closest maternity unit for their place of delivery, by groups of increasing distances between the 1st and 2nd closest maternity unit. Thus, 60.1 % of women living at less than 5 km to their closest unit chose proximity when they had to travel more than 10 km to get to the next closest unit versus 41.9 % among women whose closest unit was at 30 kilometres or more.

Figure 2 explores whether the relationship between the choice of proximity and distance between closest and next closest unit varies by distance to the closest unit. Women living at a short distance to the closest maternity unit appeared to be more sensitive to increases in distance between their first and second closest units. Thus 60.1% of women living at less than 5 km to their closest unit chose proximity when they had to travel more than 10 km to get to the next closest unit versus 41.9% among women whose closest unit was at 30 kilometres or more.

The preference for proximity, both expressed and revealed, is related to demographic and social characteristics as shown in Table 2. Younger women show a greater preference for proximity as do nullipara and women with 4 or more previous births. Household occupational status is also a determinant of choice based on proximity; women from households in the manual worker class are more likely to choose a maternity based on its proximity and also to go to the nearest unit when compared with women from households classified as being professional and managerial. Living in a rural area was associated with a higher stated preference for proximity.

**Table 2:** Maternal sociodemographic characteristics associated with expressed and revealed preference for proximity

**Sociodemographic characteristics**	**Reason for choice is proximity (NPS**^**1**^**)**		**Closest unit was chosen (VS**^**2**^**)**	
	**N**	**%**	**N**	**%**
*Maternal age*				
<25 years	1836	44.4	119470	67.5
25-29	3241	36.9	234901	62.7
30-34	3096	33.9	246679	60.5
> = 35	1478	31.1	135308	59.0
p		<.0001		<0.001
*Parity*				
0	4159	38.3	421578	61.1
1	3368	34.9	207607	63.0
2	1343	34.3	75183	63.5
3	422	34.8	19920	64.9
> = 4	247	37.3	12070	66.2
p		0.0098		<0.001
*Household socioeconomic status*				
Professional/managerial	1775	30.7	113961	52.4
Intermediate	1985	33.7	174695	59.8
Administrative, self-employed	2985	38.5	153799	63.1
Shop assistant, service workers	1370	36.5	65880	65.8
Skilled manual	839	42.4	81206	67.1
Unskilled manual	327	42.2	49376	69.9
No occupation	195	41.5	97441	65.0
p		<.0001		<0.001
*Urban/Rural*				
Urban	6196	35.7	471794	61.3
Peri-urban	2007	34.5	152067	65.3
Rural	1454	42.3	112495	60.8
p		<0.001		<0.001

^1^NPS: National Perinatal Survey.

^2^VS: Vital Statistics Registry.

The multilevel models presented in Table 3 confirm many of the associations found in our univariable analysis. Distance between the 1^st^ and 2^nd^ closest units emerges as an overriding determinant of preferences for proximity. In particular, there is a substantial jump at 30 km and over; the OR for giving proximity as a reason is at 4.62 and for selecting the closest unit is at 7.46, when compared to the distance between the two closest maternity units of 1–4 km. The association observed in Figure 2 is also confirmed in this adjusted analysis. Adjusting for distance between the first and second unit, a longer distance to the closest maternity unit is associated with a lesser preference for proximity. The more units there are in a 15 km radius, the less likely women will choose the closest unit, but this does not affect the reasons they give for their choices. Patterns are the same for expressed and revealed preference, although actual behaviour appears to be more sensitive to supply factors than reasons given by the women themselves. Furthermore, relationships with supply characteristics were very similar between models based on VS and on the NPS data.

**Table 3:** Sociodemographic and supply characteristics associated with expressed and revealed preference for proximity

**Population**	**Reason for choice is proximity (NPS**^**1**^**)**	**Closest unit was chosen (VS**^**2**^**)**	**Closest unit was chosen (NPS)**
**Level 2**			
*Constant (std err)*	−0.75 (0.11)	−0.53 (0.05)	−0.45 (0.13)
*Distance to closest maternity unit (km)*			
<5	1	1	1
5-14	0.87 (0.75-1.00)	0.95 (0.87-1.04)	0.92 (0.76-1.10)
15-29	0.68 (0.55-0.83)	0.73 (0.66-0.81)	0.62 (0.48-0.80)
30+	0.62 (0.46-0.82)	0.54 (0.49-0.60)	0.47 (0.34-0.66)
*Distance between 1st and 2nd closest maternity unit (km)*			
0	0.72 (0.63-0.83)	8.74 (8.30- 9.20)^3^	8.38 (7.07-9.93)^3^
1-4	1	1	1
5-14	1.61 (1.38-1.87)	1.63 (1.55-1.72)	1.67 (1.39-2.00)
15-29	2.70 (2.23-3.26)	3.87 (3.65-4.10)	3.29 (2.63-4.12)
30+	4.62 (3.63-5.89)	7.46 (6.91-8.06)	7.78 (5.67-10.67)
*Units in a 15 km radius*			
<4	1	1	1
4+	1.03 (0.88-1.19)	0.35 (0.32-0.40)	0.44 (0.36-0.53)
*Urban/rural*			
Urban	1		1
Peri-urban	1.06 (0.90-1.24)	0.91 (0.85-0.96)	1.05 (0.87-1.28)
Rural	1.46 (1.18-1.81)	0.85 (0.79-0.91)	0.96 (0.75-1.22)
**Level 1**			
*Maternal age*			
<25	1.28 (1.12-1.47)	1.08 (1.06-1.10)	1.17 (1.00.1.37)
25-29	1	1	1
30-34	0.94 (0.84-1.06)	0.97 (0.96-0.98)	1.00 (0.89-1.14)
35+	0.86 (0.74-1.01)	0.93 (0.91-0.95)	0.94 (0.80.1-12)
*Parity*			
0	1	1	1
1	0.89 (0.80-0.98)	1.07 (1.06.1.09)	0.99 (0.88-1.12)
2	0.89 (0.77-1.04)	1.11 (1.10-1.14)	1.14 (0.97-1.34)
3+	0.90 (0.74-1.10)	1.18 (1.15-1.22)	1.17 (0.93-1.48)
*Household socioeconomic status*			
Professional/managerial	1	1	1
Intermediate	1.06 (0.91-1.22)	1.10 (1.10-1.12)	1.23 (1.05-1.45)
Administrative, self-employed	1.20 (1.04-1.38)	1.22 (1.20-1.25)	1.35 (1.16-1.57)
Shop assistant, service workers	1.09 (0.92-1.29)	1.38 (1.35-1.41)	1.39 (1.15-1.68)
Skilled manual	1.33 (1.10-1.62)	1.45 (1.42-1.49)	1.82 (1.45-.2.28)
Unskilled manual	1.27 (0.97-1.66)	1.60 (1.56.1.64)	2.38 (1.71-3.31)
No occupation	1.39 (1.00-1.94)	1.30 (1.28-1.33)	1.56 (1.03-2.36)

^1^NPS: National Perinatal Survey.

^2^VS: Vital Statistics Registry.

^3^When units are located in the same commune, the distance between them is computed as zero; units in the same commune are all considered to be closest.

After adjusting for supply factors, maternal age and SES remain significant determinants of choice of the closest unit. This table also models actual choices using data from the NPS. Comparisons between the expressed and revealed preference models using the NPS data show that social status is a stronger predictor of actual behaviour than of the reasons given for these choices. Patterns are also similar between expressed and revealed preferences with the exception of parity which is associated with selection of the closest unit (multipara being more likely to select the closest unit), but is not related to stating that proximity is a reason for choice. To ensure that are findings were robust, the models in Table 3 on the NPS data were rerun for our sample of low risk women and the associations were very similar and are presented in an additional documentation table file (Tables 1 and 2).

**Figure 3:**
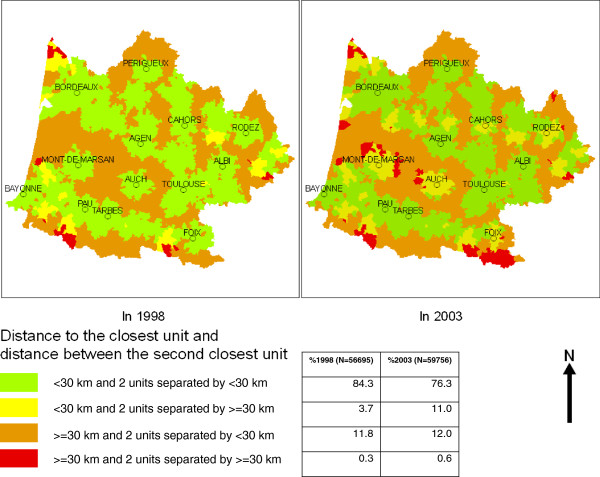
**Measuring the impact of maternity unit closures on accessibility and choice: the case of Aquitaine/Midi-Pyrénées.** The map shows the results of a case study of two French administrative regions having experienced high rates of maternity unit closures between 1998 and 2003. Regions are divided into communes where the closest maternity unit is less than 30 km away and where the 2^nd^ closest is less than 30 km away (no major constraints for accessibility or choice), and communes where the closest maternity unit is equal to or over 30 km away, but the 2^nd^ unit is within 30 km (constrained geographical accessibility, but choice possible), those where the nearest maternity unit is within 30 km, but the 2^nd^ nearest is 30 km from the first (geographical accessibility maintained, but choice constrained), and those where the closest maternity unit is 30 km away and the 2^nd^ closest is 30 km from the 1^st^ (constrained accessibility and choice).

Figure 3 illustrates how maternity unit closures can affect choice parameters in two regions in France that were strongly affected by closures between 1998 and 2003, Aquitaine and Midi-Pyrénées. The closure rates for both regions were 24.4% and 19.5%, respectively, between these two dates. This map divides the regions into communes where the closest maternity unit is less than 30 kilometres away and where the 2^nd^ closest is less than 30 km away (no major constraints for accessibility or choice), and communes where the closest maternity unit is equal to or over 30 km away, but the 2^nd^ unit is within 30 km (constrained geographical accessibility, but choice possible), those where the nearest maternity unit is within 30 km, but the 2^nd^ nearest is 30 km from the first (geographical accessibility maintained, but choice constrained), and those where the closest maternity unit is 30 km away and the 2^nd^ closest is 30 km from the 1^st^ (constrained accessibility and choice). In these regions between 1998 and 2003 geographic accessibility changed very slightly, the principal impact was on choice. Choice was constrained but geographical accessibility maintained (yellow zones) for 11.2% of births in 2003, versus 3.7% in 1998.

Our analyses focused primarily on 2003 data, because the NPS, in that year only, included a question about women’s criteria for selecting a maternity unit. To make sure that the patterns observed in our data are relevant to more recent periods, data from the most recent 2010 survey on women’s selection of the closest maternity unit were compared with 2003 (Additional file 1: documentation table file, Table 3). These analyses reveal similar patterns in 2010 and 2003.

## Discussion

In this study, we found that a determining factor of selection based on proximity was the distance between the first and second maternity unit and that the impact of this distance varied in relation to distance to the closest maternity unit and to women’s characteristics. Women already having to travel longer distances to reach their nearest maternity unit tended to travel further than other women and to choose a maternity unit for other reasons than proximity. When faced with supply constraints, multipara, younger mothers and those with a lower SES status tended to select maternity units closer to their home.

Our study has several limitations. The principal one was the difficulty of discriminating between 2 or more maternity units located within the same commune. This tended to bias results in large communes with many maternity units. Consequently, the data on rural/urban residence were hard to interpret for observed choice. This is evidenced in contradictory results that appear to suggest that women declare choosing proximity more often than the data on revealed preference actually show, especially for women living in peri-urban and in rural areas. The OR for stating proximity as the reason for choice with women in rural areas is 1.46 yet 0.85 when the actual choice is examined from the VS data (and 0.96 from the NPS data although this did not reach statistical significance). Given that our data are incapable of distinguishing between the choice of maternities in communes with 2 or more units (more frequent in urban areas), it is possible that these women living in rural areas are declaring they choose the closest unit, situated in the closest urban area. But once they have travelled far enough to get to the closest city with several maternities, they actually are free to choose between units, even if the final choice is the not the closest in absolute terms, these women are revealing a situation in which they have chosen the closest area with available units, not the unit itself. Hence the apparent contradiction between declared preference for proximity in rural areas alongside a lesser probability of actually choosing the closest unit. Furthermore, our research does not incorporate road travel time measures. Adding travel time in addition to distance would provide a more complete analysis of the way that distance affects access to care, but we are limited by the geographic zone used for this analysis (the commune) and do not feel that, given this limitation, travel distance could be interpreted adequately. In addition, calculating travel time depends on multiple hypotheses of transport mode, cost, road availability that would have rendered our analysis even more difficult to interpret at the national scale.

Another limit is that, some variables in our datasets are incomplete. Some of the French communes are not included in the NPS data and it was impossible to exclude preterm or low birthweight babies from the VS data because these data are not collected. However, by using two datasets we were able to carry out sensitivity analyses (both on the entire population, using VS data, and on a subset of low risk women in the NPS) to make sure that our results were not affected by these limitations. One difference that we did observe between our two datasets was a stronger association between our SES variable and preference for proximity in the NPS versus the VS data. A possible explanation is that the NPS SES data are better quality than the national VS data, since they are collected during an interview with the new mother. Poorer data quality may lead to misclassification and thus an attenuation of associations.

Our study focused on the choice of the maternity unit, per se, and not on the characteristics of the maternity units that were chosen. For instance, when maternity units are very similar, there may not be a real choice regarding maternity care even when two or more units are available at an “acceptable” distance from the woman's point of view. Also, the characteristics of the maternity units may affect women’s willingness to travel longer distances. Although France has a homogenous health care delivery system for maternity care, there are differences, for instance, between the public and the private sector that we did not account for here and that may impact on a woman’s choice of place of delivery. Women may also prefer to give birth in more specialised maternity units because of the level of security provided there, such as maternity units with an onsite neonatal unit [31].

Further research is necessary on these other dimensions of choice within the context of reduced maternity unit supply. For instance, integrating multiple options for care into one facility could make it possible to offset constraints imposed by maternity closures, such as facilities combining midwife led care with traditional consultant staff in Ireland [36]. Such facilities are suited to women with low-risk pregnancies – ensuring a continuity of healthcare with fewer medical interventions. They are also often considered to be more friendly environments. A subjective measure of user satisfaction with available choices could make it possible to assess the extent to which these more objective measures of choice correspond to user’s preferences [37].

This research was not designed to explore socio-cultural barriers in choice of maternity unit, but these may be an important aspect of accessibility, most notably because many French urban areas harbour large immigrant populations. These populations, mostly from North and Sub-Saharan Africa may face greater difficulties (language barriers, immigration status, discrimination) that may affect their choice. For instance, work carried out in Seine-Saint-Denis shows that country of birth was an important determinant of choice of the closest maternity unit [21], although this analysis did not consider individual social factors, which may explain some of this association. Women born outside of France constitute about one tenth of all births [38]; a study on the national level is thus not suited for an exploration of these questions.

Our data show that when women are faced with reduced supply in the area in which they reside, they will more often choose the closest maternity unit to give birth. This is especially clear in the results that show what the women actually did, which may differ to some extent to what they declared – i.e. in the revealed accessibility. This is important, because in the French context there are no restrictions on where women may deliver – notwithstanding specific individual medical and/or social situations. But unlike some other medical conditions involving hospitalization, medical care surrounding delivery is not something that can be foregone. For most women, delivery is not a planned event and being far from the maternity unit can create risks that the birth will take place before arrival at the maternity unit. A recent study carried on out-of-hospital births in the French context showed that while only about 4 in 1000 deliveries take place out of hospital in France, distance aggravates this risk for the mother and the baby [35]. Out-of-hospital delivery is associated with higher risks of adverse health outcomes for the mother and child [39,40], especially for preterm babies [41].

There was a difference between the ways that distance was incorporated into the decision making process women based on the available supply in the area where they live. The distance a woman was willing to travel to get to the nearest maternity unit seemed to be arbitrated by both absolute and relative terms. The farther the nearest maternity unit was from the woman's residence, the more likely she was to travel to get to the second closest unit. This means that a woman who had already traveled 20 km to get to a unit may be willing to travel an extra 10 km, while women living nearer to a unit would not have been willing to travel even a few extra kilometres, because this extra distance was perceived as “too far” – a sort of “willingness to travel” problem. This may reflect aspects of the arbitration process specific to choice and reveals that absolute distance may be a less important factor in this process than relative distance, conceived as time/distance trade off – part of the basis of decisions to travel people make on a daily basis, i.e. the way distance is conceived [42].

Other future research questions concern regional differences in choice and spatial accessibility to maternity units since studies have shown that reduction in the supply of maternity units has a greater impact on areas situated between two administrative regions [43] and specific spatial configurations (such as particularly isolated areas, defined in previous research as the closest maternity unit being over 45 km from the commune of residence, which is the case for 1% of births in France in 2003 [11]), where accessibility is greatly compromised.

Individual-level SES factors were important for the choice process. Increasing travel distance had a greater effect on less educated, socially disadvantaged women. There is a clear SES gradient in ability, willingness and/or desire to travel farther for care, possibly also arbitrated by other issues such as affordability (for instance transport and childcare costs). This situation may have the potential to increase SES differences in outcome if more affluent women choose more desirable or higher quality maternity units. For women of lower social standing, resorting to proximity may be less of a choice than a constraint. If this is true, it is an important factor to consider in the debate on devising and publishing hospital quality indicators to encourage decision-making, that will inform this particular population of women over others, and may increase disparities in access to place of birth.

Our analyses lead us to propose a simple indicator (2^nd^ nearest maternity unit 30 or more km from the commune of residence) for assessing the impact of maternity unit closure on the choice set of women in a given region. Our example from Aquitaine/Midi-Pyrénées illustrated that while geographic accessibility was largely preserved in this region where many maternities closed there was an important impact on choice, a consequence likely to affect user experiences and constraints. While this indicator does not capture all dimensions of choice, as discussed above, it does provide a simple and easily implementable measure to be presented together with other indicators of geographical accessibility.

## Conclusions

Taking changes in supply into consideration and how these affect choice is an important element for assessing the real impact of maternity unit closures on pregnant women’s experiences, a hotly debated subject in France and in many other countries.

In this paper, we have suggested an easily reproducible method for assessing this and we have highlighted how supply factors interact with the social characteristics of users. Our results suggest that a reduction in potential accessibility – i.e. reduced choice – is a distinct concept to spatial accessibility (distance) and should be included in evaluations of maternity unit closures.

Finally, we have described the strong relationship between maternity unit supply and women’s choices of a maternity unit using an indicator measuring preference for proximity. This indicator is readily available from routine data sources in France and could thus be used to model the effects of maternity unit closures on the choices available to the population, a useful complement to current approaches which focus almost exclusively on geographic accessibility. Similar data could be used to build indicators in other European or developed countries where routine VS data are available.

## Methods

### Data

We used two data sources: the French National Perinatal Survey (NPS) from 2003 and Vital Statistics Registry records (VS) from 2003.

### National Perinatal Surveys

All French maternity units participate in these surveys, which are undertaken periodically with the aim of monitoring perinatal health and health care practices. The survey includes all births during one week. For each birth, the survey collects information about the mother's social and demographic characteristics, healthcare utilization, as well as medical data about the delivery and the newborn. Information is also collected on her place of residence recorded at the level of the district (*département*) and in the most recent survey, the municipality (*commune)*. The data come from an interview after delivery and from medical files. We use data from the NPS which took place in October 2003. The methodology for these surveys has been described elsewhere [44,45].

The 2003 survey included N = 14 737 births. Of these, 10 378 (71%) had information relating to their commune of residence. Commune of residence was missing because this question, added for the first time to the French NPS, was not completed in all units. An analysis of missing data found no differences between the demographic and social characteristics of women with and without missing data with the exception of women who were not of French nationality (slightly higher proportions of missing data: 31.5% versus 27.8%; Women with high risk pregnancies (preterm birth, neonatal and maternal transfer to an intensive care unit) had higher proportions of missing data. To ensure that these missing cases did not create bias in our analyses, we were able to compare some results from our sample to VS data and these are presented as part of our analyses.

In the study, women were asked: “what were your two main reasons for choosing where you delivered?” The interviewer then noted the responses given. This information on the 1^st^ reason for choice was missing for 354 women (3.4%) for whom information on the place of residence was available.

Other information used for this analysis included information on the profession of the mother and the father (referred to hereafter as household socioeconomic status, or SES) and divided into 6 main categories as defined by the National Institute for Statistics and Economic Studies (INSEE): 'Professional/managerial', 'Intermediate', 'Administrative/self-employed, 'Shop assistant/service workers', 'Skilled manual', 'Unskilled manual' and 'No occupation'. We also used individual-level data on income, employment status and level of education.

We included in the analysis all women with a singleton pregnancy (N = 9657). We also identified a group of low-risk women to validate our results from the total population. High risk women are often referred to their maternity unit for delivery during pregnancy and this may affect their choice patterns. Pregnant women were considered low risk if they delivered a baby at term (37 weeks of gestation and over) with normal birthweight (2500 g and over).

### Vital Statistics Registry data

We used the 2003 data on births according to commune of residence and of birth, provided by INSEE. The commune is the lowest level of administrative division in France. In 2003 there were 36 565 communes in metropolitan France. Annual statistics for births are drawn from birth certificates completed at the vital statistics office in the commune where the birth occurred. These records also include information on maternal age, parity and SES variable as defined above. The analysis of the VS data was carried out on singleton births only, born and residing in metropolitan France (736 358 in 2003).

It was not possible to exclude preterm or low birthweight babies since data on gestational age and birthweight are not collected on the birth certificate [46].

### Distance measurements

3Distance calculations were made with a geographic information system (ArcGIS 9.3). We geocoded the location of each maternity unit open in 2003 and of each birth from the VS data and the NPS data, placing them on the map at the centre of the commune. We calculated the distances between the relevant communes in kilometres with distances computed taking into account the major regional road networks using road data from the French National Geography Institute (Institut Géographique National, IGN Route120®) with the ArcGIS Network Analyst package.

### Maternity care supply measures and geographic context

#### Measures of supply and accessibility of maternity units

To characterise the supply of maternity services with respect to each commune of residence, we computed the following measures (1) distance to the nearest maternity unit (2) the distance between the closest and next closest maternity unit and (3) the number of maternity units in a 15 km radius around the commune of residence. The 15 km radius was chosen because three-quarters of all women give birth within 15 km of their home in France [27]. Distances within the same commune were 0.

### Rural/urban

INSEE defines an urban commune as a commune or a group of communes that includes a built-up area of at least 2000 inhabitants where no building is farther than 200 m away from its nearest neighbour. In addition, more than half the population of each commune must reside in this built-up area. Accordingly, all other communes are considered rural [47].

Furthermore, we used the INSEE classification of urban and rural (ZAUER, or “zonage en aires urbaines et aires d'emploi de l'espace rural”) which divides the entire French territory according to decreasing urban character to define rural, peri-urban and urban areas [35].

### Regions experiencing high maternity unit closures

We used VS data from 1998 to identify regions which had experienced high closure rates in the 5 years preceding our study and computed supply and accessibility variables in these regions following the methods described above. Data on closures in France between 1998 and 2003 and the consequences for accessibility are reported elsewhere [11]. For this analysis, we selected two regions which had experienced high rates of closures in order to study the impact of these supply changes on our indicators of choice.

### Measures of choice

We used two measures of choice: women’s expressed and revealed preferences for proximity. We measured expressed preference for proximity based on the first response to the question asked about their principal reasons for their choice of maternity unit in the NPS. Possible responses to this question were ‘proximity’, ‘referred by a doctor or midwife’, ‘medical security’, ‘the maternity unit’s approach to childbirth’, ‘recommended by family and friends’, ‘no choice possible’ and ‘other’. We created a variable grouping together women that reported that ‘proximity’ was the main reason for their choice.

We measured women’s revealed preferences for proximity by analyzing their actual choices, i.e. whether they delivered in the maternity unit closest to home. For each birth in the VS data and in the NPS study, we created a variable measuring whether the maternity unit of delivery was the maternity unit which was closest in distance to the place of residence. Because our minimum geographic zone is the commune, we cannot measure revealed preference for proximity when there is more than one maternity unit in a commune. When there were several choices, both choices were considered the ‘closest’. This situation occurs for approximately 120 000 births in this sample (16.3% of the VS data). This limitation leads to an overestimation of the proportion of women who go to the nearest unit in communes with more than one maternity unit.

### Analysis

We first described women’s preferences for proximity and their actual choice of proximity with respect to the supply of maternity services within the commune of residence, including distance to the first unit, the relationship between the 1^st^ and 2^nd^ unit and supply density within a 15 km radius. Because the changing distance between the first and second maternity unit emerged as the key correlate of choice, we modeled this variable in more detail with respect to both revealed and expressed preference for proximity and supply characteristics of the district of residence.

Because our NPS data are from 2003 (our period of interest since we were initially investigating the effect of closures over 1998–2003 and because the question on the reasons why women chose their maternity unit was added to the 2003 survey), we checked that no substantial changes in expressed choice for these characteristics had changed in the latest available NPS (2010). Results were very similar and are presented in the Additional file 1: table file, Table 3.

We then described expressed and revealed preferences for proximity by SES characteristics. As part of this analysis, four dimensions of SES were explored using the data from the NPS (described above). The associations for these different variables were very similar and we decided to use occupational status of the household because this variable was available in both the NPS and the VS data, thereby enabling comparisons. To estimate the adjusted effects of supply and individual determinants on the choice for proximity we used multilevel logistic regression to take into consideration the hierarchical structure of our data (group level = communes, individual level = deliveries). Models were run for expressed preference using NPS data and for revealed preference using both VS and NPS data. We also reran the models for revealed preference using the NPS data on low risk deliveries in order to test the robustness of our models. Results were very similar and they are not presented here.

Finally, we assessed how our indicators of revealed preference for proximity could be applied to an analysis of trends over time by assessing change between 1998 and 2003 in a case study of two regions of France (Aquitaine and Midi-Pyrénées) experiencing high proportions of maternity unit closures. We used a framework measuring both restricted accessibility (defined as the closest maternity unit being 30 or more kilometers away) and restricted choice (defined as the 2^nd^ closest maternity unit being 30 km or more from the closest unit) to capture the effects of maternity unit closures.

## Abbreviations

SES: Socioeconomic status; IGN: Institut Géographique National (French National Geographic Institute); INSEE: Institut National de la Statistique et des Etudes Economiques (French National Institute for Statistics and Economic Studies); NPS: National Perinatal Survey; VS: Vital Statistics Registry; ZAUER: Zonage en Aires Urbaines et Aires d'Emploi de l'Espace Rural (Urban area and Rural employment area Zoning).

## Competing interests

No competing interests.

## Authors’ contributions

HP participated in the study design and drafted the manuscript. JZ conceived of the study, and participated in its design and coordination and helped to draft the manuscript. BB helped in the design and coordination of the study. ND performed the statistical analysis and provided the tables and figures. All authors read and approved the final manuscript.

## Supplementary Material

Additional file 1 **Annex tables – Comparison between low risk and all women (NPS data).** Table 1: Supply factors associated with expressed and revealed preferences for the closest unit; Table 2: Sociodemographic characteristics with expressed and revealed preferences for the closest unit, Table 3: Sample characteristics in revealed preferences for the closest unit in 2003 and 2010. Click here for file
