# Supplementary material for: Choice in maternity care: associations with unit supply, geographic accessibility and user characteristics
Source: Int J Health Geogr. 2012 Aug 20;11:35. doi: 10.1186/1476-072X-11-35 (PMC3517366; doi:10.1186/1476-072X-11-35)
Supplement: Additional file 1 — Annex tables – Comparison between low risk and all women (NPS data). Table 1: Supply factors associated with expressed and revealed preferences for the closest unit; Table 2: Sociodemographic characteristics with expressed and revealed preferences for the closest unit, Table 3: Sample characteristics in revealed preferences for the closest unit in 2003 and 2010. [file 1476-072X-11-35-S1.doc]

**Annex tables – Comparison between low risk and all women (tables 1 and 2) and sample characteristics in revealed preferences in 2003 and 2010 (table 3); National Perinatal Survey (NPS) data**

Table 1: Supply factors associated with expressed and revealed preferences for the closest unit

|  | Reason for choice is proximity  (NPS low risk[[1]](#footnote-2)) | | Reason for choice is proximity  (NPS all: all singleton[[2]](#footnote-3) ) | |
| --- | --- | --- | --- | --- |
|  | N | % | N | % |
|  | 8874 | 36.9 | 9657 | 36.4 |
| *Distance to the closest maternity unit* |  |  |  |  |
| <5 | 4020 | 38.0 | 4405 | 37.7 |
| 5-14 | 2370 | 37.4 | 2571 | 36.8 |
| 15-29 | 1904 | 34.6 | 2049 | 34.2 |
| 30+ | 578 | 34.4 | 630 | 33.5 |
| p |  | 0.05 |  | 0.0197 |
| *Distance between 1st and 2nd closest maternity unit* |  |  |  |  |
| 0 | 3561 | 25.5 | 3891 | 25.1 |
| 1-4 | 1924 | 32.4 | 2089 | 31.8 |
| 5-14 | 1866 | 42.8 | 2028 | 42.5 |
| 15-29 | 975 | 57.6 | 1051 | 56.9 |
| 30+ | 546 | 69.8 | 596 | 69.8 |
| p |  | <0.001 |  | <0.001 |
| *Units in a 15 km radius* |  |  |  |  |
| 0 | 2483 | 34.6 | 2680 | 34.0 |
| 1 | 1595 | 55.9 | 1727 | 55.7 |
| 2 | 1235 | 31.7 | 1356 | 31.6 |
| 3 | 3561 | 25.8 | 1045 | 25.1 |
| 4-9 | 1072 | 31.3 | 1167 | 31.0 |
| 10+ | 1540 | 35.6 | 1682 | 35.3 |
| p |  | <0.001 |  | <0.001 |
| *Units in a 15 km radius* |  |  |  |  |
| 0 | 2483 | 34.6 | 2680 | 34.0 |
| 1 | 1595 | 55.9 | 1727 | 55.7 |
| 2 | 1235 | 31.7 | 1356 | 31.6 |
| 3+ | 3561 | 31.7 | 3894 | 31.3 |
| p |  | <0.001 |  | <0.001 |
| *Units in a 15 km radius* |  |  |  |  |
| 0 | 2483 | 34.6 | 2680 | 34.0 |
| 1 | 1595 | 55.9 | 1727 | 55.7 |
| 2 | 1235 | 31.7 | 1356 | 31.6 |
| 3 | 949 | 25.8 | 1045 | 25.1 |
| 4 | 327 | 27.8 | 355 | 28.2 |
| 5 | 271 | 29.9 | 297 | 30.0 |
| 6 | 172 | 29.1 | 187 | 28.9 |
| 7 | 176 | 35.2 | 196 | 33.7 |
| 8 | 66 | 42.4 | 67 | 43.3 |
| 9 | 60 | 40.0 | 65 | 36.9 |
| 10+ | 1540 | 35.6 | 1682 | 35.3 |
| p |  | <0.001 |  | <0.001 |

**Table 2: Sociodemographic characteristics** associated with expressed and revealed preferences for the closest unit

|  | Reason for choice is proximity  (NPS low risk[[3]](#footnote-4)) | | Reason for choice is proximity  (NPS all: all singleton[[4]](#footnote-5) ) | |
| --- | --- | --- | --- | --- |
|  | N | % | N | % |
|  | 8874 | 36.9 | 9657 | 36.4 |
| *Maternal age* |  |  |  |  |
| <25 | 1659 | 45.2 | 1836 | 44.39 |
| 25-29 | 2984 | 37.2 | 3241 | 36.9 |
| 30-34 | 2869 | 34.2 | 3096 | 33.85 |
| >=35 | 1356 | 31.6 | 1478 | 31.06 |
| p |  | <0.001 |  | <.0001 |
| *Parity* |  |  |  |  |
| 0 | 3771 | 39.0 | 4159 | 38.33 |
| P | 3147 | 35.1 | 3368 | 34.86 |
| 2 | 1261 | 35.0 | 1343 | 34.25 |
| 3 | 376 | 33.5 | 422 | 34.83 |
| >=4 | 213 | 36.2 | 247 | 37.25 |
| p |  | 0.004 |  | 0.01 |
| *SES* |  |  |  |  |
| Professional/managerial | 1665 | 31.1 | 1775 | 30.7 |
| Intermediate | 1866 | 34.3 | 1985 | 33.7 |
| Administrative, self-employed | 2740 | 38.9 | 2985 | 38.46 |
| Shop assistant, service workers | 1246 | 37.2 | 1370 | 36.5 |
| Skilled manual | 747 | 43.0 | 839 | 42.43 |
| Unskilled manual | 293 | 42.3 | 327 | 42.2 |
| No occupation | 157 | 40.8 | 195 | 41.54 |
| p |  | <0.001 |  | <.0001 |
| *Urban/Rural* |  |  |  |  |
| Urban | 5668 | 36.0 | 6196 | 64.32 |
| Semi-urban | 1865 | 35.0 | 2007 | 65.47 |
| Rural | 1341 | 43.3 | 1454 | 57.7 |
| p |  | <0.001 |  | <0.001 |

**Table 3: Sample characteristics in revealed preferences for the closest unit in**

**2003 and 2010**

|  | 2003 | | 2010 | |
| --- | --- | --- | --- | --- |
|  | N | % closest unit  was chosen | N | % closest unit  was chosen |
|  | 9909 | 62.3 | 13711 | 62.1 |
| *Distance to closest unit* |  |  |  |  |
| <5 | 4609 | 63.1 | 6408 | 62.3 |
| 5-14 | 2633 | 63.5 | 3618 | 61.1 |
| 15-29 | 2055 | 62.0 | 2776 | 64.5 |
| 30+ | 612 | 53.0 | 909 | 56.6 |
|  |  |  |  |  |
| *Distance between 1st & 2nd unit* |  |  |  |  |
| 0 | 3778 | 80.8 | 5170 | 75.8 |
| 1-4 | 2553 | 33.4 | 3732 | 36.4 |
| 5-14 | 1954 | 51.9 | 2594 | 53.9 |
| 15-29 | 1034 | 72.3 | 1331 | 78.4 |
| 30+ | 590 | 86.4 | 884 | 89.1 |
|  |  |  |  |  |
| *Units in a 15 km radius* |  |  |  |  |
| <4 | 6916 | 70.1 | 69.07 | 69.0 |
| 4+ | 2993 | 44.4 | 4241 | 46.6 |
|  |  |  |  |  |
| *Urban/rural residence* |  |  |  |  |
| Urban | 6448 | 61.9 | 8856 | 624 |
| Peri-urban | 2021 | 66.2 | 2883 | 63.1 |
| Rural | 1440 | 58.7 | 1972 | 59.2 |
|  |  |  |  |  |
| *Maternal age* |  |  |  |  |
| <25 | 1893 | 68.9 | 2319 | 67.1 |
| 25-29 | 3296 | 62.7 | 4506 | 63.6 |
| 30-34 | 3164 | 59.6 | 4174 | 60.1 |
| 35+ | 1525 | 58.8 | 2574 | 58.4 |
|  |  |  |  |  |
| *Parity* |  |  |  |  |
| 0 | 4238 | 62.1 | 4771 | 60.7 |
| 1 | 3443 | 61.2 | 4624 | 61.9 |
| 2 | 1388 | 64.1 | 1945 | 62.6 |
| 3+ | 709 | 66.6 | 1045 | 64.3 |
|  |  |  |  |  |
| *SES* |  |  |  |  |
| Professional/managerial | 1797 | 51.9 | 3,065 | 52.8 |
| Intermediate | 1999 | 60.9 | 3,187 | 62.1 |
| Administrative, self-employed | 2470 | 64.5 | 2,791 | 63.5 |
| Shop assistant, service workers | 531 | 62.7 | 605 | 63.5 |
| Skilled manual | 1391 | 65.9 | 1,750 | 65.1 |
| Unskilled manual | 1199 | 71.6 | 1,640 | 69.4 |
| No occupation | 376 | 68.6 | 526 | 72.4 |

1. NPS low risk: live singleton births with gestational age>=37 weeks and birthweight>=2500g [↑](#footnote-ref-2)
2. NPS all: all live singleton births [↑](#footnote-ref-3)
3. NPS low risk: live singleton births with gestational age >=37 weeks and birthweight>=2500g. [↑](#footnote-ref-4)
4. NPS all: all live singleton births. [↑](#footnote-ref-5)
